# Accelerated degradation of polyetheretherketone and its composites in the deep sea

**DOI:** 10.1098/rsos.171775

**Published:** 2018-04-25

**Authors:** Hao Liu, Jianzhang Wang, Pengfei Jiang, Fengyuan Yan

**Affiliations:** 1 State Key Laboratory of Solid Lubrication, Lanzhou Institute of Chemical Physics, Chinese Academy of Science, Lanzhou, People's Republic of China; 2 University of Chinese Academy of Sciences, Beijing, People's Republic of China

**Keywords:** degradation, polyetheretherketone, composites, deep sea, hydrostatic pressure

## Abstract

The performance of polymer composites in seawater, under high hydrostatic pressure (typically few tens of MPa), for simulating exposures at great depths in seas and oceans, has been little studied. In this paper, polyetheretherketone (PEEK) and its composites reinforced by carbon fibres and glass fibres were prepared. The seawater environment with different seawater hydrostatic pressure ranging from normal pressure to 40 MPa was simulated with special equipment, in which the seawater absorption and wear behaviour of PEEK and PEEK-based composites were examined *in situ*. The effects of seawater hydrostatic pressure on the mechanical properties, wear resistance and microstructure of PEEK and its composites were focused on. The results showed that seawater absorption of PEEK and its composites were greatly accelerated by increased hydrostatic pressure in the deep sea. Affected by seawater absorption, both for neat PEEK and composites, the degradation on mechanical properties, wear resistance and crystallinity were induced, the degree of which was increasingly serious with the increase of hydrostatic pressure of seawater environment. There existed a good correlation in an identical form of exponential function between the wear rate and the seawater hydrostatic pressure. Moreover, the corresponding mechanisms of the effects of deep-sea hydrostatic pressure were also discussed.

## 1. Introduction

At present, to protect the marine environment as far as possible, the use of lubricating oil or grease is greatly limited in some crucial friction systems of modern ships and marine hydraulic machineries, e.g. rudder bearings, propeller shaft bearings, guide units and shafts of turbines and hydraulic components of pumps [1–3]. On the contrary, an environment-friendly and energy-efficient seawater lubrication model is being strongly recommended and widely used in marine applications [4–6]. Compared with lubricating oil and grease, however, seawater has not only ultra-low viscosity and poor lubricating capacity but also very high corrosivity, greatly limiting the use of traditional metal/metal friction pairs [7–11]. Accordingly, research and development of novel high-performance seawater-lubricated anti-friction/anti-wear materials, which should be with good adaptability to seawater lubrication and strong resistance to seawater corrosion, are drawing more and more attention [12–16]. As a special engineering plastic with high mechanical strength, chemical inertness, high wear resistance, self-lubricating ability and good workability, polyetheretherketone (PEEK) along with its fibre-reinforced composites has been proved to be an excellent lubricating material with seawater lubrication adaptability and seawater corrosion resistance, and widely served as high-performance seawater-lubricated friction component at the normal pressure [17,18].

In recent years, with the rapid development of deep-sea exploitation, PEEK-based seawater-lubricated components are increasingly expanding towards deeper seawater environment. And accordingly, their service performance in the deep-sea environment has been paid great attention. With the increase of seawater depth, however, the hydrostatic pressure of seawater will also correspondingly increase greatly; specifically, every 100 m increase in seawater depth is associated with 1 MPa increase in the hydrostatic pressure. In some previous works, it has been reported that the seawater absorption of some thermosetting resin-based film (such as vinyl resin) was greatly improved with the increase of hydrostatic pressure, leading to a degradation in mechanical properties and microstructure [19–21]. On the other hand, however, in other works it has been found that significant improvement on both modulus and strength of some thermoplastic polymers (such as PTFE, PE and POM) can occur by applying high hydrostatic pressure on these polymers with inert kerosene or castor oil [22–24]. Moreover, it has been confirmed that the wear behaviour of several self-mated alloys was closely correlated to the seawater hydrostatic pressure [25]. Clearly, deep-sea hydrostatic pressure may have a great impact on mechanical properties, tribological behaviour and even microstructure of materials. But so far, it is still unclear that the possible changes in the properties of PEEK and PEEK-based composites with the increase of seawater hydrostatic pressure, making it difficult to achieve wide application in the deep sea.

In this paper, the deep-sea environment, which is characterized by the incorporation of seawater and hydrostatic pressure, was simulated with special equipment. Seawater absorption and wear behaviour of PEEK and PEEK composites reinforced by short carbon fibres and glass fibres were examined *in situ*. We believe that the results will benefit the fundamental understanding of the wear behaviour of thermoplastic polymers, providing theoretical and technical guidance for the design, material selection and protection of friction components serving in the deep sea.

## 2. Experimental section

### 2.1. Materials

PEEK powders (VESTAKEEP 4000FP, average particle size of 65 µm, density of 1.32 g cm^−3^) were commercially obtained from Degussa Co. Ltd (Germany). Milled polyacrylonitrile-based carbon fibres (density of 1.77 g cm^−3^, diameter of 7 µm, length–diameter ratio of 4–8) and glass fibres (density of 2.60 g cm^−3^, diameter of 13 µm, length–diameter ratio of 5–10) were provided by Nanjing Fiberglass R&D Institute (China).

316 stainless steel (UNS S31600, with a composition of 0.03% C, ≦2.0% Mn, ≦0.045% P, ≦0.03 S, ≦0.75% Si, 16–18% Cr, 10–14% Ni, 2–3% Mo, balanced Fe) was used as the counterpart material in wear tests for its excellent corrosion resistance to seawater.

### 2.2. Preparation of seawater

Artificial seawater was prepared according to ASTM D1141-98. The chemical composition of seawater is listed in table 1. The pH value of as-prepared seawater was adjusted to 8.2 using 0.1 mol l^−1^ NaOH solution.

**Table 1.** Chemical composition of artificial seawater.

constituents	concentration (g l^−1^)
NaCl	24.53
MgCl_2_	5.20
Na_2_SO_4_	4.09
CaCl_2_	1.16
KCl	0.695
NaHCO_3_	0.201
KBr	0.101
H_3_BO_3_	0.027
SrCl_2_	0.025
NaF	0.003

### 2.3. Preparation of composites

PEEK-based composites reinforced by carbon fibres (denoted as CF/PEEK) and glass fibres (denoted as GF/PEEK) with a volume fraction of 10% were prepared by hot press moulding. The fibre fraction of 10% was chosen due to its optimum balance between stiffness, toughness, thermal stability and more importantly, the tribological performance in seawater [17]. First, PEEK and fibre powders were mechanically mixed at a rotor speed of 4000 r.p.m. for 2 min. Then the mixed powders were pressed at 7 MPa in a mould and simultaneously sintered at 380 ± 2°C for 4 h. The crystallization temperature of PEEK was 303.6°C [26]. After naturally cooling below 100°C and releasing from the mould, the PEEK-based composites specimen with a dimension of Φ 120 mm × 30 mm was obtained. The specimens were then machined to specific dimensions for different tests. As control samples, neat PEEK specimens were also prepared following the same procedure.

### 2.4. Friction and wear test

**Figure 1.**
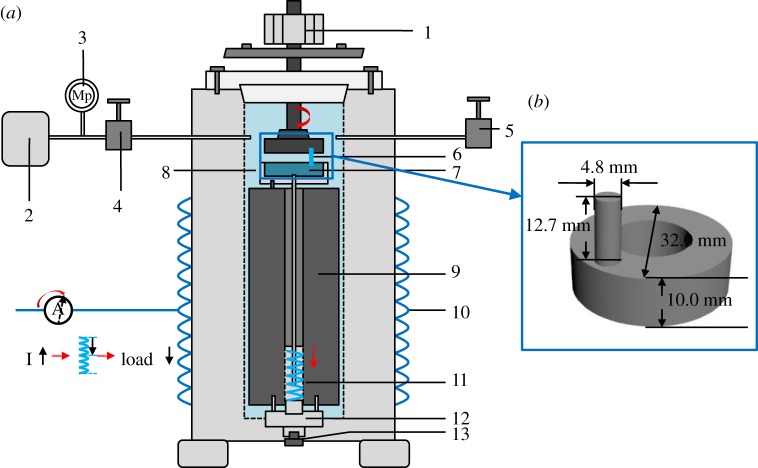
Schematic diagram of (*a*) the test apparatus: (1) rotary motor, (2) hydraulic test pump, (3) pressure gauge, (4) inlet valve, (5) outlet valve, (6) pin specimen, (7) ring specimen, (8) artificial seawater, (9) magnets, (10) metallic support, (11) magnetically driving system; (*b*) pin-on-ring friction pairs.

A specialized friction tester was developed to simulate specific seawater environments with different hydrostatic pressure ranging from normal pressure to 40 MPa, and *in situ* evaluate the friction and wear behaviour of PEEK and its composites. A schematic diagram of the test apparatus is shown in figure 1. Hydrostatic pressure was controlled by manually operated high pressure control valves, which are supplied with the valve panel assembly. Two are provided to be used with the inlet and outlet ports. The hydrostatic pressure was measured by a pressure gauge, which is attached to the top of the pressure vessel. The value of hydrostatic pressure was recorded with a pressure transducer. The pressure transducer output is 4–20 mA. The friction couple was fixed in the autoclave, which was not directly connected with outside, but driven and loaded magnetically to ensure the good sealing of the autoclave. The frictional couple, in a pin-on-disc contact mode, comprises a rotary pin with a size of Φ 4.8 mm × 12.7 mm*,* and a stationary counterpart disc with a size of Φ 32 mm × 10 mm. Prior to each test, the autoclave was filled and pressured using artificial seawater. The friction and wear tests were carried out at a linear velocity of about 0.5 m s^−1^ and a contact stress of 18 MPa for a sliding duration of 120 min. Before each test, the sliding surfaces of the pin and the disc were abraded to reach a surface roughness of about 0.10 µm and ultrasonically cleaned with acetone for 15 min. At the end of the friction and wear test, the seawater hydrostatic pressure in the autoclave was released and the pin was disassembled and ultrasonically cleaned with acetone for 15 min. Then the wear mass loss of the pin was measured using an electronic balance with an accuracy of 0.1 mg (Ohaus, Adventure™, America, no. 121240520). The wear volume loss of the pins was calculated as

2.1 $$V=\frac{\Delta M}{\rho },$$

where *V* is the wear volume loss (mm^3^), Δ*M* is the mass loss of the pin specimen (mg) and *ρ* is the density (g cm^−3^) of the pin specimen. The specific wear rate of the worn pins was calculated as

2.2 $$K=\frac{V}{d\cdot L},$$

where *K* is the wear rate (mm^3^ N^−1^ m^−3^), *d* is the sliding distance (m) and *L* is the load (N). Five repeat measurements were conducted for each test condition, and the average of wear rates was reported in this work.

### 2.5. Seawater absorption test

Seawater absorption of PEEK composites under specific hydrostatic pressure was tested by a stainless vessel integrated with a water pump. Before tests, both the vessel and the pump were filled with artificial seawater. The hydrostatic pressure of seawater ranging from atmosphere pressure to 40 MPa can be simulated with an accuracy of 1 MPa. The seawater absorption tests were conducted at different hydrostatic pressures following the standard ASTM D570. The specimens were immersed in the seawater with different hydrostatic pressures at room temperature (approx. 25°C), and weighed every 24 h to determine the weight-gain with an accuracy of 0.1 mg. The seawater absorption was expressed as the percentage of the weight-gain to the original mass of the tested specimen. The seawater absorption was calculated as

2.3 $$m_{t}=\frac{M_{t}-M_{0}}{M_{0}}\times 100\%,$$

where *m_t_* is the seawater absorption, %; *M_t_* is the weight of specimens at immersion time *t*, g; *M*_0_ is the initial weight, *g*. The weighings were repeated until the increase in weight per two period averages less than 1% of the total increase in weight or 5 mg, whichever is greater; the specimen was then considered substantially saturated. For each type polymer, the average seawater absorption of five specimens was reported.

### 2.6. X-ray diffraction measurements

X-ray diffraction (XRD) measurements were used to determine the crystallinity of PEEK and PEEK-based composites using Cu K*α* radiation (*λ* = 1.54 Å) on a PANalytical Empyrean diffractometer. The degree of crystallinity was calculated by the Hermans–Weidinger method [9].

### 2.7. Mechanical tests

Mechanical properties of PEEK and its composites after experiencing saturated seawater absorption were examined. Namely, the tensile, compression and flexural properties were evaluated using a DY35 universal materials tester (Adamel Lhomargy, France), according to ISO527-2/1A:1993, ISO604:2002 and ISO178:2001, respectively. The Izod impact strength was measured with a ZBC1400-2 impact machine (Sans, China) at a rate of 2.9 m s^−1^ in accordance with ASTM D256A. Mechanical properties were measured after the saturated samples had been fully dried at room temperature (approx. 25°C) to constant weights. The mechanical properties of PEEK and its composites unaffected by seawater absorption were also examined using the same methods. All these tests were conducted at room temperature, and an average value of at least three repeated tests was taken for each material.

### 2.8. Observation on the worn surfaces

To research how the worn surface morphology of CF/PEEK and the counterpart 316 SS changes in different hydrostatic pressure, SEM analysis was conducted by a JEM-1200EX scanning electron microscope (SEM). The accelerating voltage for the SEM observations was 20 kV and the load current was approximately 70 µA. Before observation, the worn surface of the samples was coated with a thin film of gold (JFC-1600 auto fine coater, JEOL Ltd, Japan). Three samples worn in one specific hydrostatic pressure were used. For each sample, five places on the surfaces of each sample were selected at random. SEM micrograph was taken at 1000× magnification for CF/PEEK and 500× magnification for 316 SS, and the characteristic morphology of the worn surface was clearly visible.

## 3. Results and discussions

### 3.1. Seawater absorption

**Figure 2.**
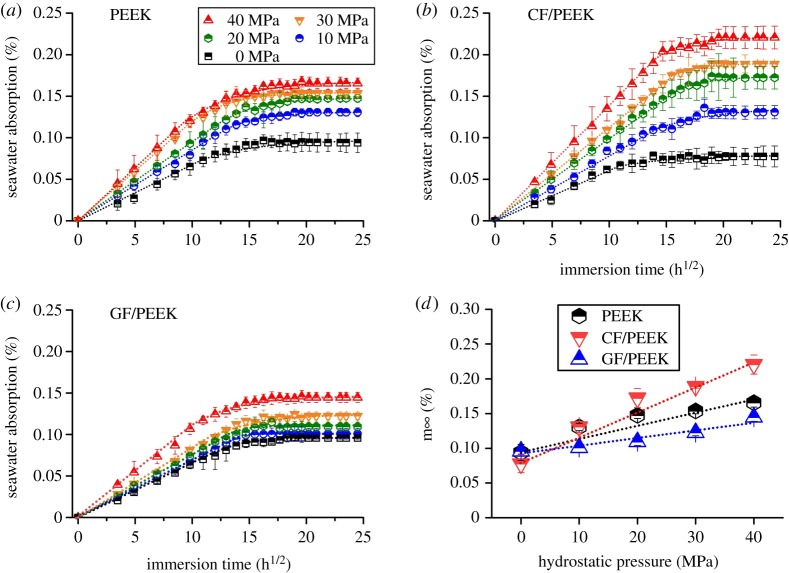
Variation in seawater absorption with immersion time (*a*–*c*) and saturated seawater absorption (*d*) of PEEK and PEEK-based composites.

The variations in seawater absorption of PEEK and its composites with the immersion time are shown in figure 2. It can be seen that, for both PEEK and its composites, seawater absorption grows linearly and rapidly with immersion time at the initial stage, then gradually slows down and finally reaches a plateau, indicating the equilibrium state of absorption. The slope of linear growth shows the rate of seawater absorption, and the absorption at the plateau corresponds to the saturated seawater absorption. Clearly, not only the seawater absorption rate but also the saturated seawater absorption is significantly increased with the increase of seawater hydrostatic pressure. Namely, the seawater absorption of PEEK and its composites can be greatly accelerated by high hydrostatic pressure in the deep sea. Moreover, compared with PEEK and GF/PEEK, CF/PEEK is more susceptible to the hydrostatic pressure. As shown in figure 2*d*, the saturated seawater absorption of CF/PEEK is lower than that of PEEK and GF/PEEK under normal pressure, but it will be much higher than those of PEEK and GF/PEEK when the seawater hydrostatic pressure exceeds 20 MPa. Once the seawater hydrostatic pressure increases from normal pressure to 40 MPa, the saturated seawater absorption of CF/PEEK will be tripled.

In fact, it has been reported in previous work that when CF/PEEK was immersed in tap water, its saturated water absorption increased from approximately 0.20% to approximately 0.25% as the hydrostatic pressure increased from 0.1 to 10 MPa [27,28]. Such accelerating effect of increased hydrostatic pressure on water absorption was ascribed to enlarged osmotic pressure of water with the increase of hydrostatic pressure, favourably promoting the penetration ability of water. In essence, the diffusion process of seawater throughout PEEK materials is governed by the difference of seawater chemical potential between the solution phase and the polymer solid phase. The equilibrium of seawater absorption corresponds to the equality of seawater chemical potentials in the two phases. The greater the difference, the faster the seawater diffuses through polymer, and accordingly the greater the saturated level of seawater absorption. Under specific seawater hydrostatic pressure, the seawater chemical potential in solution phase can be expressed as [29]

3.1 $$\mu (p)=\mu (p_{0})+RT\ln\left(\frac{p}{p_{0}}\right),$$

where *μ*(*p*) and *μ*(*p*_0_) are the chemical potentials of seawater under hydrostatic pressure *p* and normal pressure *p*_0_, respectively. Clearly, at the same ambient temperature, with the increase of seawater hydrostatic pressure, the seawater chemical potential in solution phase is accordingly increased. Before seawater absorption, there exists no seawater in the polymer phase. It means the difference of seawater chemical potential between the solution phase and the polymer solid phase will grow with the increase of seawater hydrostatic pressure, resulting in the accelerated seawater absorption. Therefore, high hydrostatic pressure in the deep sea can be considered a kind of driving force for seawater diffusion in polymer materials.

### 3.2. Mechanical performance degradation

**Figure 3.**
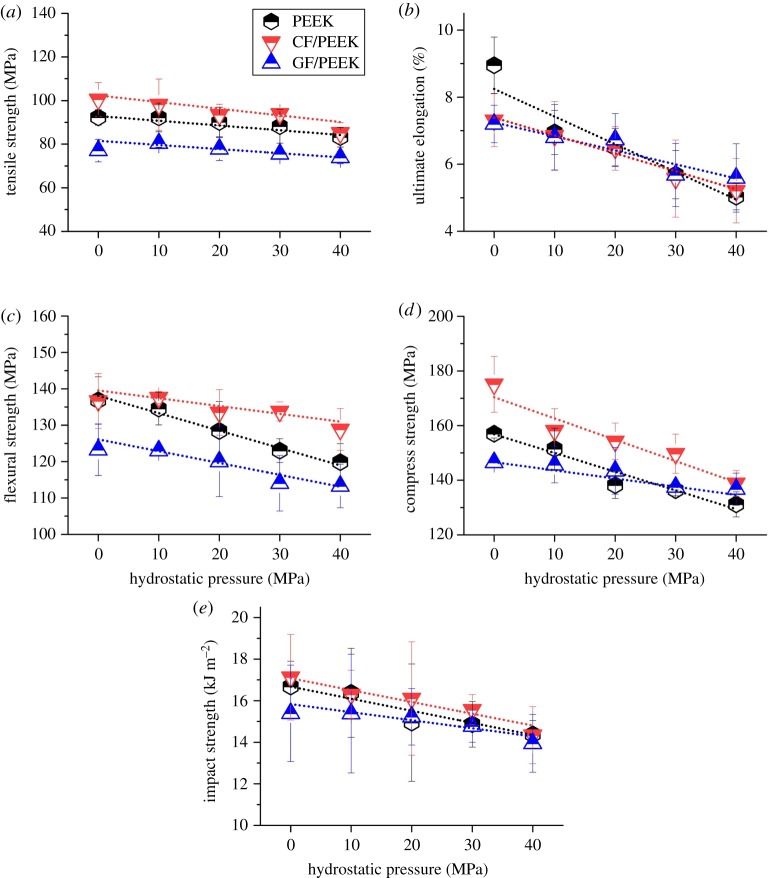
Variation in mechanical properties of PEEK and PEEK-based composites after experiencing saturated seawater absorption under specific seawater hydrostatic pressure, (*a*) tensile strength, (*b*) elongation at tensile breaking, (*c*) flexural strength, (*d*) compressive strength and (*e*) impact strength.

It is well known that diffusion of water throughout a polymer matrix may lead to changes in mechanical performance. Figure 3 shows the mechanical performances of PEEK-based materials after experiencing saturated seawater absorption under specific hydrostatic pressure. Clearly, for both PEEK and its composites, their mechanical properties, namely tensile strength, elongation at tensile breaking, flexural strength, compressive strength and impact strength, are degraded due to seawater absorption, and almost linearly decrease with the increase of hydrostatic pressure.

**Figure 4.**
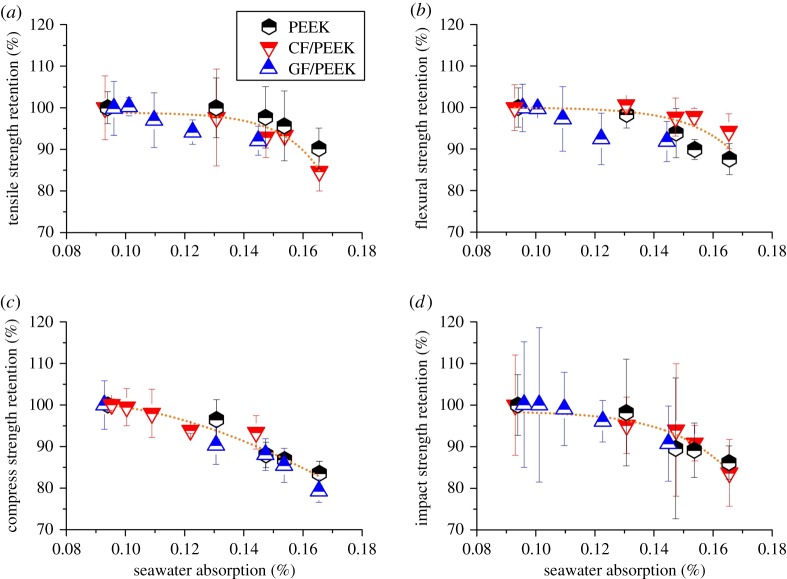
(*a*) Tensile strength retention, (*b*) flexural strength retention, (*c*) compressive strength retention and (*d*) impact strength retention of PEEK, CF/PEEK and GF/PEEK against seawater absorption, respectively.

As the mechanical tests were conducted in ambient pressure, the hydrostatic pressure may not directly influence the mechanical strengths. Instead, their reversible mechanical damage may indirectly root in the accelerated seawater absorption induced by elevated hydrostatic pressure. To verify this point, the mechanical strength retention was plotted against seawater absorption, as shown in figure 4. It can be seen that as the seawater absorption increases, the mechanical strength retention gradually decreases. Namely, mechanical strength loss is associated with the seawater absorption to some extent. The amount of seawater absorption may serve as a useful indication of the loss of mechanical strength.

Mechanical degradation of polymer materials induced by water absorption is generally attributed to the plasticizing or swelling effect of absorbed water [30,31]. Because of the invasion of water molecules, the interaction between macromolecular chains as well as the fibre/matrix combination was greatly weakened, leading to the loosened texture and the degraded mechanical performance. Such process can be called plasticization or swelling, which is primarily a diffusion-controlled process and thus apparently depends upon the amount of absorbed water. However, not all absorbed water molecules make a contribution to the reversible degradation of the mechanical properties of PEEK and its composites. Because of the macromolecular structure containing moderately polar ketone group, PEEK has been proved to be able to bind water by hydrogen bridges [32]. IR and MRS analyses by several authors previously indicated that only water connected to macromolecules by hydrogen bonds can cause irreversible changes in properties of PEEK materials [33,34].

### 3.3. Wear behaviour

**Figure 5.**
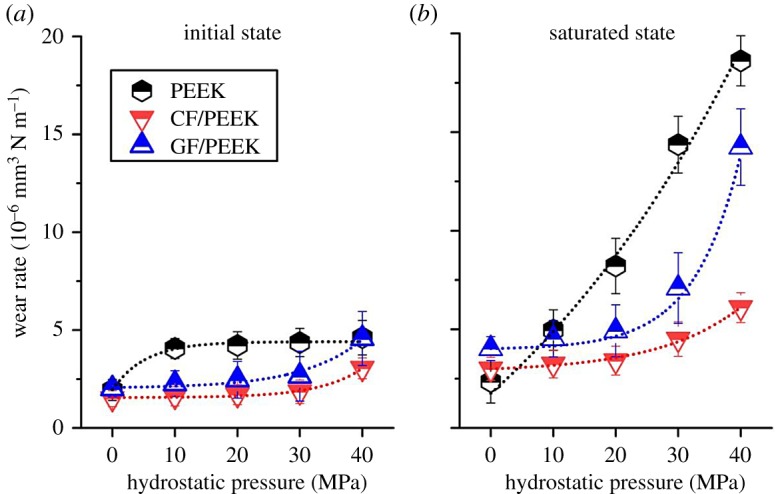
Variations in wear rates of PEEK and its composites in different states of seawater absorption, (*a*) initial state and (*b*) saturated state, with the seawater hydrostatic pressure.

Figure 5 shows the correlation between seawater hydrostatic pressure and the wear rates of PEEK and its composites in different states of seawater absorption.

Clearly, the wear behaviour of both PEEK and its composites is greatly influenced by the degree of seawater absorption. In seawater with specific seawater hydrostatic pressure, if PEEK and its composites have undergone saturated absorption prior to friction tests, they may exhibit higher wear rates than those with no prior seawater absorption. The seawater absorption-induced acceleration of wear loss becomes increasingly larger, especially under the hydrostatic pressures in excess of 20 MPa. Whether affected by prior saturated seawater absorption or not, however, both PEEK and its composites show increased wear rates with the increase of hydrostatic pressure. And much faster growth in wear rate is exhibited for the materials previously affected by saturated seawater absorption. And more interestingly, regardless of PEEK, GF/PEEK or CF/PEEK, there exists a good correlation between the wear rate and the seawater hydrostatic pressure, and the corresponding fitted equations are listed in table 2. Clearly, the correlation can be described in a form of exponential function as follows:

3.2 $$K_{p}=K_{0}\exp\left[a+b\left(\frac{p}{p_{0}}\right)+c{\left(\frac{p}{p_{0}}\right)}^{2}\right],$$

where *K_p_* is the wear rate under specific seawater hydrostatic pressure, mm^3^ N^−1^ m^−1^; *K*_0_ is the wear rate under normal pressure, mm^3^ N^−1^ m^−1^; *p* is the seawater hydrostatic pressure, MPa; *p*_0_ is the normal pressure, about 0.1 MPa; and *a*, *b*, *c* are constants.

**Table 2.** Fitted parameters of the correlation between wear rate and seawater hydrostatic pressure.

		*W*_0_ (10^−6^ mm^3^ N^−1^m)	*a*	*b*	*c*	*R*^2^
initial state	PEEK	1.9	1.1 × 10^−3^	1.0 × 10^−3^	−1.3 × 10^−6^	0.970
	CF/PEEK	1.5	3.5 × 10^−2^	−1.1 × 10^−3^	6.7 × 10^−6^	0.921
	GF/PEEK	2.0	7.2 × 10^−3^	3.1 × 10^−4^	4.2 × 10^−6^	0.985
saturated state	PEEK	2.3	−7.7 × 10^−4^	8.6 × 10^−4^	−8.1 × 10^−7^	0.998
	CF/PEEK	3.0	1.1 × 10^−4^	−1.6 × 10^−5^	4.8 × 10^−7^	0.988
	GF/PEEK	4.0	5.6 × 10^−4^	−1.6 × 10^−5^	1.1 × 10^−10^	0.989

Apparently, the tribological performance of polymeric material is not inherent but positively associated with its mechanical strength. That is one of the reasons that further weakened wear resistance was observed on samples in the saturated state at specific hydrostatic pressure.

The accelerated wear loss of PEEK and its composites with the elevated hydrostatic pressure can be reasonably attributed to the seawater absorption growth induced by elevated hydrostatic pressure. It has been confirmed that absorbed seawater leads to the decrease in mechanical performance of PEEK materials, and the degree of mechanical degradation is positively correlated with the degree of seawater absorption. And such degradation in mechanical properties is a key factor that leads to the increase in wear loss. During the wear process, the seawater absorption is a dynamic self-accelerated process. It has been confirmed that absorption of seawater started from the surface, resulting in the plasticization or softening of the sliding surface of PEEK materials in water [29]. And the softening effect may be further increased due to the attack of massive ions in seawater on carbonyl group –CO–, contained in PEEK molecular structure, as reported by Stolarski [35]. Such softening of sliding surface was considered to be responsible for higher wear rate of PEEK. The corroded surface was worn off more easily, generating a fresh sliding surface more beneficial to the invasion of seawater molecules. And then a vicious circle is induced, that is, seawater absorption of sliding surface → softening of sliding surface → accelerated abrasion of corroded surface → generating a fresh sliding surface → accelerated seawater absorption of sliding surface → more serious softening of sliding surface → accelerated abrasion of corroded surface, etc.

The addition of carbon or glass fibre considerably improved the wear resistance of PEEK, because fibres can bear a great portion of the applied stress on the sliding surface to reduce the damage to the polymer matrix direct interaction between polymer and metal [36,37]. Moreover, compared with GF/PEEK composite, CF/PEEK composite exhibits better wear resistance, because carbon fibre has higher strength and better chemical stability than glass fibre. The constituents of glass fibre (mainly SiO_2_, etc.) produce their hydroxides and hydrates in water, and the mechanical strength of these was lower than that of SiO_2_ [38,39].

**Figure 6.**
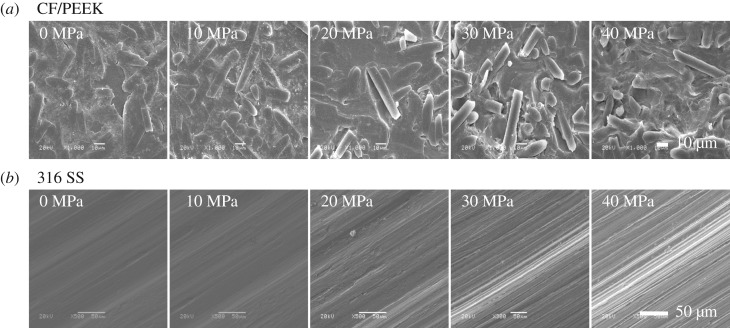
Morphologies of the worn surfaces of CF/PEEK (*a*) and counterpart steel (*b*) sliding in seawater with different hydrostatic pressures.

Figure 6 shows the morphology of the worn surfaces of CF/PEEK composite and counterpart steel sliding in different seawater environments, in which the effect of seawater hydrostatic pressure on the wear behaviour can be visually revealed. The worn surface of CF/PEEK becomes increasingly rough along with the increase of hydrostatic pressure, and is characterized with more significant peeling off of PEEK matrix or drawing out of carbon fibres, revealing increasingly serious wear loss. And similar to CF/PEEK, the counterpart steel also shows a worn surface increasingly roughening with the increase of hydrostatic pressure, also reflecting the aggravated wear. Evidently, such changes in worn surfaces are well consistent with the aforementioned variation trends of wear rates with hydrostatic pressure.

### 3.4. Microstructure characterization

**Figure 7.**
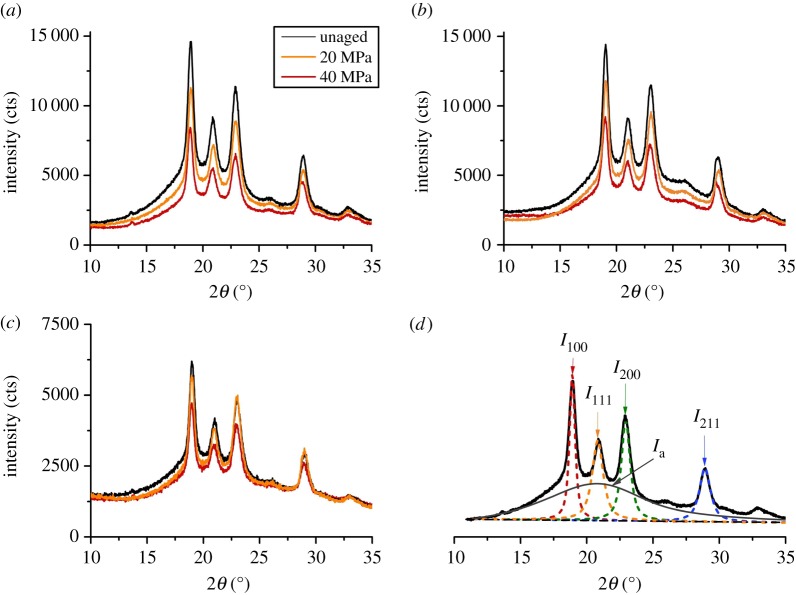
XRD patterns of (*a*) PEEK, (*b*) CF/PEEK, (*c*) GF/PEEK affected by saturated absorption in seawater with different hydrostatic pressures; (*d*) schematic diagram of XRD curve spalling and peaking method for calculating the crystallinity of PEEK matrix (XRD pattern of neat PEEK without any treatment).

It has been found that accelerated degradation on the mechanical properties and wear resistances of PEEK and its composites are induced by increased hydrostatic pressure in the deep sea, indicating possible microstructure transformation induced by increased hydrostatic pressure. Figure 7 shows the XRD pattern of PEEK and its composites after being immersed in seawater with different hydrostatic pressures and reaching saturated absorption.

It can be seen in figure 7*a* that neat PEEK presents main reflection peaks located at 2*θ* position of 18.8°, 20.7°, 22.9° and 28.9°, corresponding to the scattering of the (110), (111), (200) and (211) lattice planes of the orthorhombic unit cell [26,40]. The pattern of the PEEK composites is qualitatively similar to that of neat PEEK, merely with wider and less intense peaks. Clearly, crystallites in the PEEK matrix are not irreversibly swollen by the diffusing seawater because no distinct shift in the angular position of the reflection peaks is found and thus the spacing of the inter-chain is unaltered by seawater. Based on the XRD patterns, a quantitative analysis of the crystallinity of neat PEEK and its composites was conducted as shown in the schematic diagram in figure 7*d*, and the results are listed in table 3. Clearly, once immersed in seawater with specific hydrostatic pressure and reached saturation, both neat PEEK and its composites show the decrease in crystallinity, and the decrease degrees gradually increase with the increase of hydrostatic pressure.

**Table 3.** The crystallinity of the PEEK matrix by XRD analysis.

ageing	crystallinity (%)		
environment (MPa)	PEEK	CF/PEEK	GF/PEEK
unaged	45.3	39.5	32.7
0	44.8	38.5	30.1
10	43.2	37.7	30.1
20	42.8	37.5	29.8
30	41.7	37.2	29.0
40	40.9	36.9	28.5

As is well known, PEEK is a semi-crystalline polymer, consisting of the crystalline phase and the amorphous phase. It has been found that water tends to diffuse through the amorphous phase, and the crystalline regions are often assumed to be not affected by absorbed water. Based on the XRD analyses in this work, however, it is evident that the crystalline regions are also affected by absorbed water, resulting in the decrease in the degree of crystallinity. As the seawater penetrates, intermolecular hydrogen bonds in PEEK matrix are forced open and attached by available hydroxyl groups in water molecules. This would cause the associated crystalline structure to collapse into an amorphous region because the size of the side groups including water molecules is too great. Similar phenomenon was reported in the case of poly (vinyl alcohol) by Iwamoto *et al*. [41–43]. But after all, the decrease in the crystallinity can lead to the degradation in the mechanical strength and wear resistance of polymer materials, this has been fully proved in many previous studies [44–47].

## 4. Conclusion

(i) The seawater absorptions of PEEK and its composites were greatly accelerated by high seawater hydrostatic pressure in the deep sea, resulting in growing absorption rate and saturated absorption.
(ii) Affected by saturated seawater absorption, PEEK and its composites showed irreversible degradation in mechanical strengths. Owing to the accelerating effect of increased hydrostatic pressure on seawater absorption, the degree of mechanical degradation for PEEK and its composites was increased nearly linearly with the increase of hydrostatic pressure.
(iii) Regardless of PEEK, GF/PEEK or CF/PEEK, a growth in wear rate was shown with the increase of seawater hydrostatic pressure, which was in an identical form of exponential function. Much faster growth in wear rate was exhibited for the materials previously affected by saturated seawater absorption.
(iv) Affected by saturated seawater absorption, both neat PEEK and its composites exhibited a slight decrease in crystallinity, and the decrease in degrees gradually increased with the increase of seawater hydrostatic pressure.

## Data accessibility

Our data are deposited at Dryad: (http://dx.doi.org/10.5061/dryad.kr8t0) [48].
